# Pre‐Exposure to Chemicals Increases Springtail Vulnerability to High Temperatures

**DOI:** 10.1111/gcb.70374

**Published:** 2025-07-23

**Authors:** Micha Wehrli, Jian Ge, Stine Slotsbo, Martin Holmstrup

**Affiliations:** ^1^ Department of Ecoscience Aarhus University Aarhus Denmark; ^2^ Department of Environmental Chemistry Swiss Federal Institute of Aquatic Science and Technology – Eawag Dübendorf Switzerland

**Keywords:** climate change, Collembola, *Folsomia candida*, multiple stressors, pesticides, soil arthropods, thermal death time, thermal stress

## Abstract

Global climate change is increasing the frequency and intensity of heat waves, posing a significant threat to ectothermic organisms. Concurrently, chemical pollution, including heavy metals and pesticides, remains a pervasive environmental stressor. This study investigates the effects of sub‐lethal copper and fluazinam exposure on the thermal tolerance of the soil‐dwelling springtail, *Folsomia candida* . Using a thermal death time (TDT) framework, we assessed how pre‐exposure to these toxicants at two acclimation temperatures (20°C and 24°C) influenced survival under heat stress. Our findings indicate that toxicant exposure reduced heat tolerance at moderately high temperatures (32.5°C) but had negligible effects at extreme temperatures (37°C). Acclimation at 24°C mitigated the negative effects of both toxicants, suggesting an enhanced capacity for cellular homeostasis under warm conditions. Additionally, soil type influenced thermal tolerance, highlighting the importance of environmental context in multiple stressor interactions. These findings highlight the need to integrate realistic thermal exposure scenarios in ecotoxicological assessments to improve predictions of organismal vulnerability under climate change.

## Graphical abstract

This study shows that exposure to common pollutants like copper and fungicides makes springtails more vulnerable to high temperatures. However, when the springtails were gradually adapted to warmer conditions, their heat tolerance improved, even with chemical exposure. These findings suggest that pollution and climate change together may pose a greater risk to soil organisms than either stressor alone.

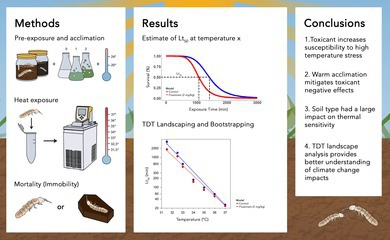

## 1 Introduction

Anthropogenic global warming has already reached 1.5°C above the pre‐industrial levels, and temperature will likely continue to rise in the coming decades (IPCC 2022). Along with the rising average temperature, current models and observations show that the frequency and intensity of heat waves have increased, as will diurnal and seasonal peak temperatures (IPCC 2022). Recent studies highlight increases in air temperature, heatwave intensity, and duration (Perkins‐Kirkpatrick et al. 2024), which is projected to further increase soil temperatures, predicted to outpace air temperatures (García‐García et al. 2023; Lembrechts et al. 2022). Such major changes in our climate compel societies and ecosystems across the globe (Richardson et al. 2023).

In ectothermic organisms, the temperature of the environment determines metabolic and physiological rates (Willmer et al. 2000). In the “permissive” range of temperatures, the organism can maintain cellular homeostasis allowing growth, development and reproduction at rates following a unimodal relationship with temperature (Ørsted et al. 2022). At temperatures beyond the permissive range, this balance is interrupted, and the rate of cell injury due to thermal stress becomes greater than the rate of cellular repair mechanisms (“Homeostatic capacity rate”), thus interrupting cellular homeostasis. The longer an organism is outside its species‐specific permissive range, cellular injuries will accumulate until the moribund organism is not able to recover from the accumulated damage and will perish (Ørsted et al. 2022). In a recent report, Jørgensen et al. (2022) demonstrated that the rate at which heat‐related cellular damages accumulate doubles with every 1°C across more than 100 ectothermic animal species. On the basis of this broad analysis, the authors warned that heat mortality in the future may become much more common, with drastic consequences for the abundance and diversity of aquatic and terrestrial ectothermic animals.

In addition to extreme temperature, unintended pollution with toxic chemicals such as pesticides and heavy metals is a pervasive threat to biodiversity and ecosystem functioning (IPBES 2019). Moreover, synergistic interaction between the effects of toxicants and extreme temperature can exceed the expected additive effects (Holmstrup et al. 2010; Orr et al. 2020). This phenomenon has been termed toxicant‐induced climate susceptibility (TICS) or climate‐induced toxicant susceptibility (CITS) by several authors (Delnat et al. 2019; Hooper et al. 2013; Moe et al. 2013; Verheyen et al. 2019; Verheyen and Stoks 2019, 2020) and recently has been shown in experiments with various ectothermic organisms (J. Ge et al. 2023; Liess et al. 2016; Verheyen and Stoks 2023). Synergistic interactions may be due to high temperatures increasing the internal concentrations of the toxicant via altered toxicokinetics (Dai et al. 2021; Wehrli, Slotsbo, Fomsgaard, et al. 2024) or be based on one stressor exacerbating the effect of the other via toxicodynamic interactions (Holmstrup et al. 2014).

Understanding the mechanisms involved and the magnitude of such combined stressor effects is important for realistic predictions of the ecological consequences of climate change and chemical pollution. However, thorough analyses of toxicant‐induced susceptibility to heat stress are urgently missing. Thus, most TICS studies have tested thermal tolerance at only one or a few stressful temperatures and exposure durations, ignoring that thermal injury accumulation depends on both stress intensity (i.e., the temperature) and exposure time (Cossins and Bowler 1987) and providing only an incomplete picture of the potential effects arising from TICS. Using a thermal death time framework (TDT) circumvents this shortage by providing the specific relationship between temperature and exposure time of a species (Rezende et al. 2014) and allowing for the prediction of the accumulation of injuries in dynamic thermal systems (Jørgensen et al. 2021). Hence, the TDT approach tests the dose–response relationship between survival and exposure time at multiple static temperatures ranging from mildly stressful (“permissive”) temperatures to acutely lethal temperatures. This allows for calculation of doses (i.e., exposure times at a certain temperature) of thermal injury causing 50% mortality (Lethal time; Lt_50_). Regression analysis of Lt_50_ values against exposure temperature provides the TDT landscape from which the temperature dependence of thermal injury accumulation rate can be derived (Ørsted et al. 2022).

In the present TICS study, we explored how the TDT relationship of springtails is affected when they are pre‐exposed in soil microcosms to environmentally realistic concentrations of copper or fluazinam (a fungicide) at two different acclimation temperatures. Our study used the common soil‐living springtail, *Folsomia candida* (Willem, 1902). This model species is widely used in physiological, ecotoxicological, and ecological studies (Fountain and Hopkin 2005). Springtails are found in almost every soil type and biome and have ecological relevance because of their contribution to decomposition and nutrient cycles in soil by grazing on microorganisms and detritus (Potapov et al. 2023; Rusek 1998). We also note that *F. candida* is found where the soil temperature is projected to increase and reach high temperatures relevant for our study during heat waves (GBIF 2025).

In two previous studies, we mapped the TDT landscape of several springtail species and showed that acclimation temperature is important for the rate of thermal injury accumulation (Wehrli, Slotsbo, Ge, and Holmstrup 2024; Xie et al. 2023), as has been shown for other ectotherms (Dallas and Rivers‐Moore 2012; Geerts et al. 2015; Gunderson and Stillman 2015). However, the influence of toxicants on the TDT or the interaction between toxicants and acclimation temperature has not previously been investigated. We aimed to answer two main questions: Firstly, will pre‐exposure to a toxicant lead to a change, for example, an increase in the thermal susceptibility of springtails? Secondly, does acclimation temperature influence the effect of toxicants on thermal susceptibility?

## 2 Materials and Methods

### 2.1 Test Animals

*Folsomia candida* (‘Berlin strain’) was obtained from a mass population cultured in our laboratory since 1994 (Simonsen and Christensen 2001). Mass cultures were raised in Petri dishes with a base of moist plaster of Paris/active charcoal (8:1 w:w). Cultures were kept at constant 20°C (12:12 h light/darkness cycle) and were fed *ad libitum* with dried Baker's yeast. Adult springtails (20–30 days old) of similar size were used for all experiments.

### 2.2 Copper Contaminated Soil

We wanted to pre‐expose springtails to realistic and sub‐lethal concentrations of copper and therefore collected an agricultural soil contaminated by Cu from a former timber preservation factory at Hygum (Jutland, Denmark), where the soil Cu concentrations have been well‐characterized (Scott‐Fordsmand et al. 2000). The soil type at this field site was sandy clay, and it had not been treated with pesticides for at least 20 years, and no other contaminants than Cu were found at the site (Nielsen et al. 2015; Pedersen et al. 1999). Soil from the upper 20 cm layer was dug out from three areas representing ‘low’ (background level), ‘medium’, and ‘high’ levels of Cu contamination. The soil was dried at 80°C for 48 h prior to the determination of the Cu concentration and sifted through a 2 mm mesh. Approximately 0.5 g of the soil sample in each sampling spot was added to glass vials, followed by 2 mL of 68% nitric acid. The vials were heated in a heating block where the temperature gradually increased from 80°C to 120°C in 3 h and remained at this temperature until the content in the vials became transparent (digested). Digested samples were diluted to 5 mL using 5% nitric acid and placed at room temperature overnight. The supernatant was transferred to 5 mL plastic centrifuge tubes, and the copper concentration was measured using inductively coupled plasma‐optical emission spectrometry with an accuracy of ±0.1 mg/kg (5800 ICP‐OES, Agilent, Germany). Calibration was performed using copper calibration standards (Cu Pure standard, 1000 μg mL^−1^, PerkinElmer, USA). The efficiency of the digestion was verified by analyzing certified reference material (oyster tissue material from the National Institute of Standards and Technology, US Department of Commerce, and lobster hepatopancreas from National Research Council Canada). The measured concentration was approximately 95% of the certified values. The Cu concentrations of the ‘low’ (control), ‘medium’, and ‘high’ Cu contaminated soils were 21.3 ± 1.5, 437 ± 18.7, and 689.9 ± 21.8 mg Cu/kg^−1^ dry mass (mean ± SE; *n* = 3). In a previous study, we showed that these Cu levels did not cause mortality in 4‐week microcosm tests with *F. candida* (Ge et al. 2023).

### 2.3 Fluazinam Contaminated Soil

As for copper, we wanted to pre‐expose springtails to a soil concentration of fluazinam that is realistic (Hakala et al. 2020) and still in the sub‐lethal range of concentrations that we have reported in a previous study (Wehrli, Slotsbo, Fomsgaard, et al. 2024). To this end, we used LUFA 2.2 soil, a standard sandy loam obtained from LUFA Speyer (Speyer, Germany), for the fluazinam exposure. The spiking procedure was done as described by Wehrli, Slotsbo, Fomsgaard, et al. (2024) and Wehrli, Slotsbo, Ge, and Holmstrup 2024. In brief, fluazinam (Sigma Aldrich, Darmstadt, Germany; CAS 79622–59‐6, ≥ 98% purity) was dissolved in acetone (180 mL kg^−1^ dry soil) to obtain 0, 2, and 10 mg kg^−1^ dry mass soil. Realistic environmental concentrations up to 2.1 mg kg^−1^ have been found in agricultural soils (Hakala et al. 2014). The soil was mixed vigorously, spread out in a glass tray, and placed in a fume hood to evaporate the acetone for at least 24 h before use. The actual start concentrations of fluazinam and hydroxylfluazinam in test soil were measured using LC–MS/MS as described in Wehrli, Slotsbo, Fomsgaard, et al. (2024) and Wehrli, Slotsbo, Ge, and Holmstrup (2024) and were found to be 1.21 ± 0.03 and 7.39 ± 0.19 mg kg^−1^ dry soil (mean ± SE, *n* = 3), respectively. The soil concentrations of hydroxylfluazinam at the start of the experiment were found to be 0 ± 0, 0.021 ± 0.001, and 0.066 ± 0.001 mg/kg soil.

### 2.4 Pre‐Exposure to Contaminated Soil

The dry test soils were moistened by adding 266 mL (Cu polluted soils from Hygum) and 221 mL de‐ionized water (fluazinam‐spiked LUFA 2.2 soil) kg^−1^ dry mass, respectively, providing optimal humidity levels for springtails. 50 g of moistened test soil was added to 200 mL glass beakers with air‐tight screwcaps. About 100 animals were added to each beaker. Twice per week, the beakers were briefly opened for aeration and for feeding the springtails with 2 mg dried Baker's yeast. For each test soil, we established 50 beakers, of which half were incubated at 20°C ± 0.2°C (“control acclimation”) and the other half at 24°C ± 0.2°C (“warm acclimation”). These acclimation temperatures represent normal and warm summer temperatures for *F. candida* in their natural habitats and are not stressful (Ge et al. 2023; Roeben et al. 2023; Wehrli, Slotsbo, Fomsgaard, et al. 2024). Springtails were exposed to the test soils for 3 weeks and then used for thermal tolerance assays. For each test soil (Hygum and LUFA), acclimation temperature (20°C and 24°C), and concentration of chemical (three copper levels in Hygum soil; three fluazinam concentrations in LUFA soil), we exposed roughly 2500 adult specimens for the thermal tolerance assays. We did not assess mortality during the pre‐exposure to chemicals in this study, but our previous results of experiments using the same soil types and chemicals have shown that the chemical exposures were below concentrations where mortality occurs (> 1600 mg Cu kg^−1^ Hygum soil; > 10 mg fluazinam kg^−1^ LUFA soil) (Ge et al. 2023; Wehrli, Slotsbo, Fomsgaard, et al. 2024).

### 2.5 TDT Assays

To generate the TDT relationship, the springtails were exposed to static thermal tolerance assays for increasing exposure times to generate logistic dose–response curves (survival versus exposure time) for each exposure temperature. This was done to define how much time is needed to reach 50% mortality (Lt_50_) at each respective temperature (i.e., a dose of thermal stress).

Springtails were extracted from the test soil by adding tap water to the beaker and gently stirring the contents with a small spoon. Because of the hydrophobic cuticle of the springtails, they would, within a few minutes, float on the surface and were scooped with a spoon and placed on a Petri dish with dry plaster of Paris to remove excess water. After that, the springtails were transferred to a Petri dish with moist plaster of Paris to prevent evaporative water loss until they were used for thermal tolerance assays. Flotation is a standard procedure in research involving springtails (OECD 2016), and it was done consistently across all treatments, allowing comparisons of the effects of the two soil contaminants and acclimation temperature. The time elapsed from extraction to testing of thermal tolerance was less than 1 h.

We used static assays with a temperature range from 31.5°C to 37°C (31.5°C, 32.5°C, 33°C, 34°C, 35°C, 36°C, and 37°C ± 0.04°C). The temperatures used in this experiment represent realistic (although extreme) high soil temperatures experienced by *F. candida* (Lembrechts et al. 2022). 20 to 30 springtails were randomly selected and transferred with an aspirator to a 2 mL centrifuge tube. To maintain saturated humidity in the tubes, we placed a moistened filter paper disc (5 mm diameter) in the lid before closing the tube. Subsequently, at least 10 tubes with springtails for each acclimation temperature (20°C and 24°C) and test soil (fluazinam contaminated LUFA soil: 0 (control), 2 and 10 mg kg^−1^; copper contaminated Hygum soil: ‘Low’ (21 mg kg^−1^, control), ‘medium’ (437 mg kg^−1^) and ‘high’ (690 mg kg^−1^)) were submerged in a water bath with the desired temperature. On the basis of a previous TDT study of *F. candida* (Wehrli, Slotsbo, Ge, and Holmstrup 2024), we tentatively distributed the sampling times at each exposure temperature to ensure that the full dose–response curve could be fitted to observations. Thus, at the highest tested temperature, sampling times were in the range 0 to 120 min, whereas at the lowest tested temperatures in the range 0 to 3000 min (Figure S1). At each sampling time, one tube of each treatment combination was removed from the water bath, and the springtails in the tubes were immediately transferred to separate Petri dishes with moistened plaster of Paris (diameter 35 mm). After recovery at 20°C for 24 h, the animals were scored as survivors if they walked in a normal coordinated manner after stimulation with a fine paintbrush. The remaining animals were scored as dead. As a control for a possible effect of confinement in a closed centrifuge tube, we tested the survival of springtails (3 replicates of 30 animals) at 20°C after confinement for 3, 5, and 7 days, respectively, and observed full survival at all durations.

**FIGURE 1:**
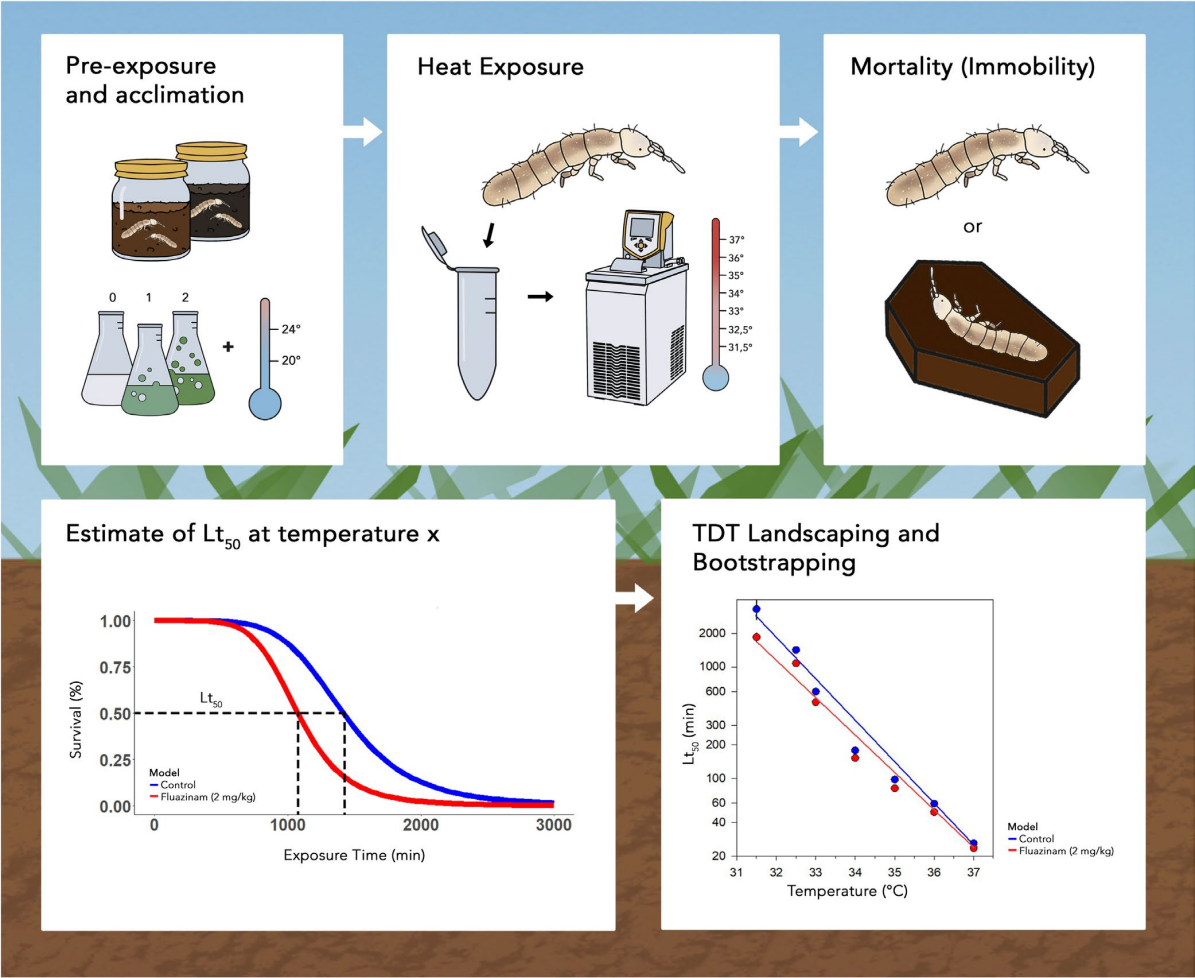
Schematic outline of the experimental principle.

The water baths used (Lauda ECO RE 1050, Lauda, NJ, USA) were accurate to 0.02°C. Water temperature was measured with Tinytag data loggers equipped with PB‐5001 thermistors (Tinytag, Chichester, UK). The temperature inside the tubes reached the water bath temperature within 60 s, as documented with a fine copper‐constantan thermocouple, connected to a Grant Squirrel 1250 datalogger (Grant Instruments, Cambridge, UK). The overall experimental principle is summarized in Figure 1.

### 2.6 Concentration of Fluazinam and Metabolites in Springtail Tissue

With the purpose of validating exposure via soil and investigating the influence of acclimation temperature, we measured fluazinam and selected metabolites in springtail tissues. At the end of the pre‐exposure in soil, we collected about 40 springtails per sample and determined their combined fresh mass using a Sartorius SC 2 microbalance (Sartorius AG, Goettingen, Germany). Fluazinam and metabolites were extracted from springtail tissue by adding 500 μL of acetonitrile and a metal bead (diameter: 3.2 mm) to a 2 mL Eppendorf tube with springtails and homogenizing the sample in a Geno/grinder (SPEX SamplePrep, Metuchen, NJ, USA) for 1 min. Homogenized samples were then ultrasonicated at 60°C for 45 min, followed by 10 min of centrifugation at 16,602 *g*. Subsequently, the supernatant was transferred to a clean amber glass vial. The pellet was then redissolved with 500 μL of acetonitrile, and the procedure was repeated. Finally, 500 μL of the combined supernatants was concentrated under a constant flow of nitrogen and resuspended in 100 μL of 25% acetonitrile before liquid chromatography with tandem mass spectrometry (LC–MS/MS) analysis as described in detail by Wehrli, Slotsbo, Fomsgaard, et al. (2024) and Wehrli, Slotsbo, Ge, and Holmstrup (2024). The limit of detection (LoD) and limit of quantification (LoQ) were reported as the lowest measurable standard resembling 0.024 ng mL^−1^; all samples below this threshold were considered as below LoD and set to 0. The internal concentrations were reported as ng mg^−1^ fresh body mass, and the soil concentrations as mg kg^−1^ dry weight soil.

## 3 Data Analysis

The data analysis was conducted using R Studio version 2023.09.1 + 494 (R Studio Team 2023) with R version 4.3.2. (R Core Team 2023). The data analysis was performed using the same R‐script as in previous work (Wehrli, Slotsbo, Ge, and Holmstrup 2024). Survival was calculated as the relative fractions of live animals compared to the total organisms in each sample.

Dose–response analysis was performed for the entire dataset using a 2‐parameter log‐logistic model:

(1) $$f\left(t\right)=\frac{C}{1+\exp\;\left(b\left(\log\;\left(t\right)-\log\left(a\right)\right)\right)}$$

where *f(t)* represents the survival fraction at time *t* (min), *C* is the survival in the control (at *t* = 0), *b* is the slope, and *a* is Lt_50_ (min). The weight was set to the total number of exposed organisms per sample, and the type was set to binomial distribution, with upper and lower limits fixed at 1 and 0, respectively, using the R package drc (Ritz et al. 2015). The experimental approach with exposure of animals in centrifugation tubes submerged in a temperature‐controlled water bath, followed by recovery at room temperature for 24 h before scoring of survival fraction, did not allow repeated observations, which would secure monotonic decreasing survival over time (Figure 1). However, logistic regression analysis can easily handle non‐monotonicity in data as long as observation times (exposure times) include start values of (almost) full survival and (almost) full mortality to cover the complete dose–response curve.

**FIGURE 2:**
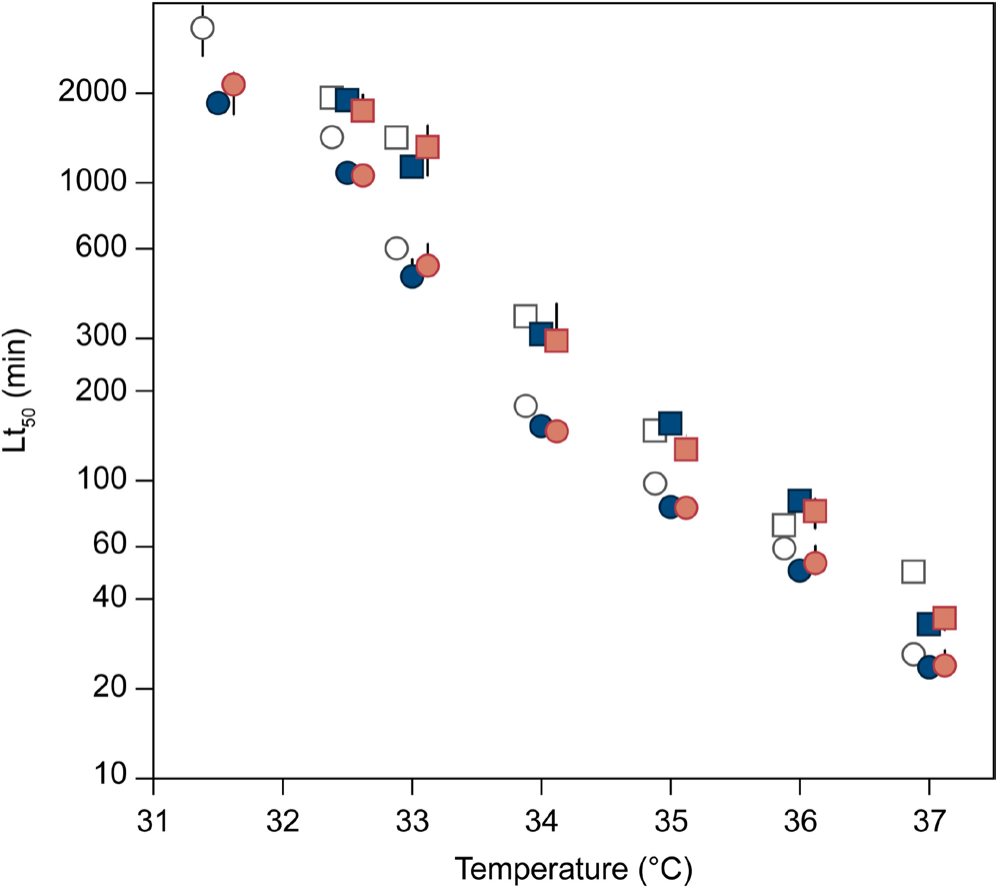
Thermal death time (TDT; the time taken to reach 50% mortality) of adult *Folsomia candida*. 356 plotted against exposure temperature. Before TDT assays, animals were pre‐exposed to LUFA soil contaminated with fluazinam at either 20°C (circles) or 24°C (squares). TDT values were calculated in minutes (see text for further explanation). Error bars represent SE; where not seen, the SE is hidden by the symbol. White symbols: Control soil (no contaminant), blue symbols: 2 mg fluazinam kg‐1 dry soil, red symbols: 10 mg fluazinam kg‐1. Note the log‐scale on the *y*‐axis.

**FIGURE 3:**
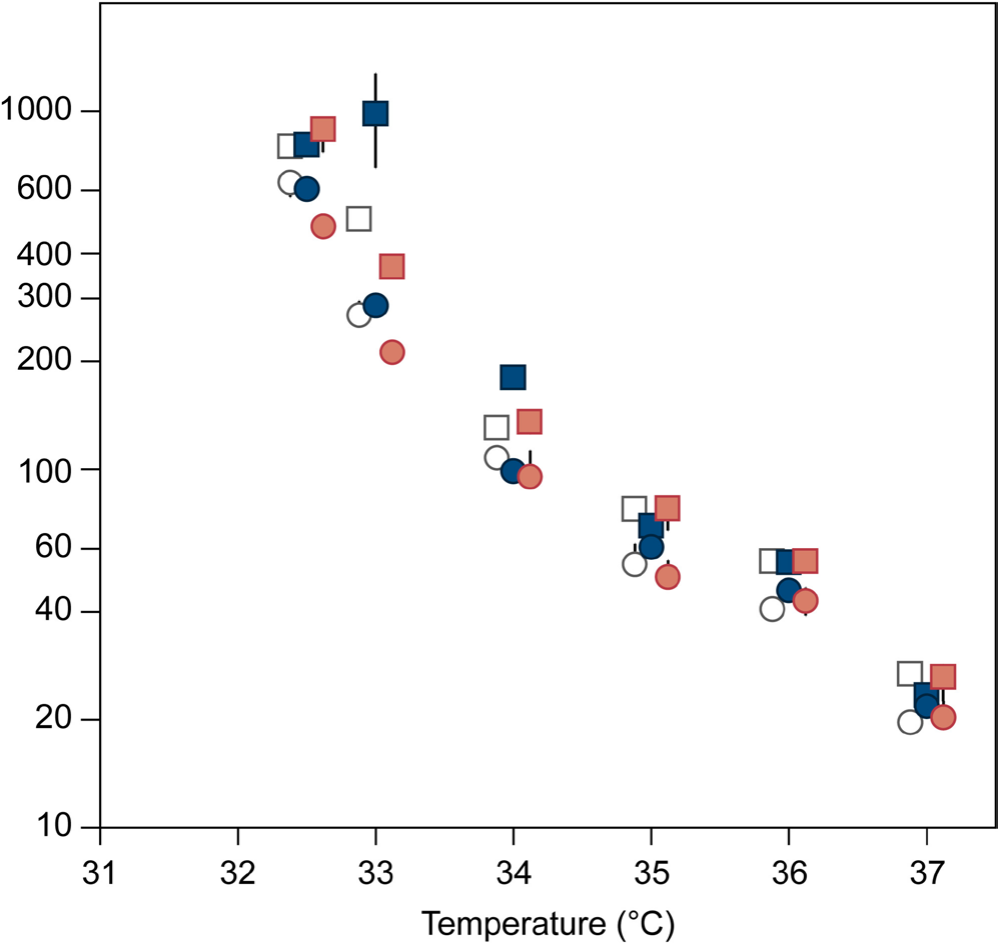
Thermal death time (TDT; the time taken to reach 50% mortality) of adult *Folsomia candida* plotted against exposure temperature. Before TDT assays, animals were pre‐exposed to Hygum soil contaminated by copper at either 20°C (circles) or 24°C (squares). TDT values were calculated in minutes (see text for further explanation). Error bars represent SE; where not seen, the SE is hidden by the symbol. White symbols: Control soil (no contaminant), blue symbols: Medium copper, red symbols: High copper. Note the Log‐scale on the *y*‐axis.

The extracted means and standard errors of Lt_50_ from all 75 dose–response models (see data in Figures 2 and 3) were re‐sampled 500 times, assuming normal distribution using bootstrapping in R. The re‐sampled Lt_50_ were log transformed and fitted with linear models against the exposure temperature. The mean and 95% confidence intervals of the regression slopes, intercepts, and Lt_50_ at 32.5°C and 37°C, respectively, were calculated accordingly. These two specific temperatures were chosen to show the lowest temperature where we obtained a complete set of dose–response curves in all treatments and at the highest tested temperature. We interpreted overlapping confidence intervals between treatments as indications that the effects were not statistically significant.

For analysis of body concentrations of fluazinam and hydroxy fluazinam, the dataset was checked for normality with a Shapiro–Wilk test and Levene's test. Afterward, ANOVA was performed to compare the effect of exposure concentration, the effect of acclimation temperature, and their interaction.

## 4 Results

### 4.1 Body Concentrations of Toxicants

The internal concentration of the fungicide and its metabolite in the springtails after 3 weeks of exposure significantly depended on the exposure concentration but not the acclimation temperature (Table 1). In the case of the parent compound fluazinam, we found that acclimation temperature did not show significance (ANOVA: *p* = 0.22); in contrast, we observed an increase in internal concentrations between exposure concentrations (ANOVA, *p* = 0.002). Similarly, we found an increase in the hydroxy‐metabolite concentration with exposure concentration (ANOVA, *p* < 0.001) but not for acclimation temperature (ANOVA *p* = 0.14). The interaction between temperature and exposure concentration was not significant for any of the soil concentrations. Furthermore, we found elevated hydroxy metabolite concentrations compared to the parent compound concentrations by a factor of 36 to 71 (Table 1). The secondary metabolite's concentration, sulfhydryl‐fluazinam, was negligible and is not reported here. It should be noted that “internal concentrations” include compounds associated with the cuticle wax layer as well as body lipid compartments.

**TABLE 1:** Concentration of fluazinam and hydroxyl‐fluazinam (the primary metabolite of fluazinam) in springtail bodies after exposure for 3 weeks in soil spiked with fluazinam.

Temperature	Nominal concentration of fluazinam in soil (mg kg^−1^ dry soil)	Body concentration (ng mg^−1^ fresh mass) (mean ± SE)	
		Fluazinam	Hydroxyl‐fluazinam
20°C	2.0	0.01 ± 0.01	0.36 ± 0.02
	10.0	0.05 ± 0.01	2.11 ± 0.50
24°C	2.0	0.01 ± 0.01	0.50 ± 0.06
	10.0	0.04 ± 0.01	2.83 ± 0.15

*Note:* The LoD was 0.024 ng mL^−1^ before transformation to ng mg^−1^ fresh mass.

The body Cu concentration of springtails was not determined in the present study. However, a previous study has shown that *F. candida* exposed for 3 weeks in Hygum soils is able to efficiently regulate and maintain the same internal concentration at about 45 mg Cu kg^−1^ dry mass in the Cu‐contaminated soils as in the control soil used in the present study (Ge et al. 2023).

### 4.2 TDT in Soil Contaminated With Fluazinam

**FIGURE 4:**
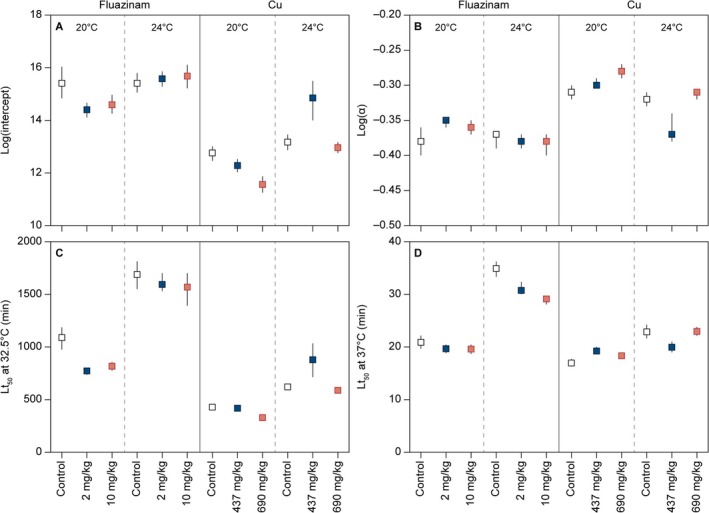
The intercepts (A) and slopes of regression lines fitted to TDT data (B) as well as the estimated Lt_50_ at 32.5°C (C) and Lt_50_ at 37.0°C (D) of adult *Folsomia candida* . Before TDT assays, animals were pre‐exposed to LUFA soil contaminated with fluazinam or Hygum soil contaminated by copper at either 20°C or 24°C. Error bars represent 95% confidence intervals. If the 95% confidence intervals were not overlapping between estimates, they were interpreted as a statistically significant difference.

Survival‐time data fitted logistic relationships with good credibility (Figure S1). Linear regressions between Log(Lt_50_) and temperature gave statistically significant relationships, with data shown in Figure 2 and parameters shown in Figure 4 and Table S1. The Lt_50_ at 32.5°C (Lt_50‐32.5°C_) of springtails acclimated at 20°C in uncontaminated soil was about 1100 min (Figure 4). Contamination with fluazinam resulted in significantly lower Lt_50_ values (about 800 min; Figure 4). The Lt_50_ was not different when comparing 2 mg and 10 mg fluazinam kg^−1^. The Lt_50_ at 37°C for animals acclimated at 20°C was similar (ca. 20 min) in all three treatments and not influenced by pre‐exposure to fluazinam (Figure 4). When animals were acclimated at 24°C, the Lt_50_ at 37°C was significantly higher in control soil; however, the effect size was less than 3 min (Figure 4D).

Although both Lt_50‐32.5°C_ and slope of the regression were significantly highest (numerically) in the control soil after pre‐acclimation at 20°C, the effect of fluazinam disappeared when springtails were pre‐acclimated at 24°C (Figures 2 and 4; Table S1).

### 4.3 TDT in Soil Contaminated With Copper

As for fluazinam‐polluted soil, survival‐time data fitted logistic relationships with good credibility (Figure S1). Linear regressions between Log(Lt_50_) and temperature gave statistically significant relationships, with data shown in Figure 3 and parameters shown in Figure 4 and Table S1. The Lt_50_ at 32.5°C of springtails acclimated at 20°C in uncontaminated Hygum soil was about 400 min (Figure 4). This value was significantly higher than for animals pre‐exposed to the highest soil copper concentration, but not different from the medium copper concentration (Figure 4; Table S1). The Lt_50_ at 37°C was similar (ca. 20 min) in all three treatments and not influenced by pre‐exposure to copper‐polluted soil.

Although both Lt_50‐32.5°C_ and slope of the regression were significantly highest (numerically) in the control soil after pre‐acclimation at 20°C, the negative effect of copper disappeared when springtails were pre‐acclimated at 24°C (Figures 3 and 4; Table S1).

### 4.4 Effect of Acclimation Temperature on TDT

Acclimation at a higher temperature (24°C vs. 20°C) had a clear positive effect on the thermal tolerance with almost 50% higher Lt_50‐32.5°C_ values in both soil types and at all concentrations of fluazinam and copper (Figures 2, 3, 4; Table S1). However, at the highest temperatures tested, the positive effects of warm acclimation were less clear (Figures 2 and 3).

### 4.5 Effect of Soil Type on TDT

Soil type had a clear effect on thermal tolerance. A comparison of uncontaminated soils across acclimation temperatures showed that animals pre‐exposed in LUFA soil were much more heat tolerant than animals pre‐exposed in Hygum soil (Figures 2, 3, 4; Table S1).

## 5 Discussion

As an answer to the first of our research questions, our results indicate that the sub‐lethal effects of fluazinam, manifested in the high internal concentration of hydroxyl‐fluazinam, did indeed increase the susceptibility of *F. candida* to high temperatures. In this study, we used a laboratory culture that has been maintained in our laboratory since 1994 (Simonsen and Christensen 2001). Laboratory culturing could result in adaptations to laboratory conditions and perhaps loss of phenotypic plasticity in thermal susceptibility; however, it has been shown in other species that such changes are often negligible (Maclean et al. 2018). Since our study takes a comparative approach, we believe our results are relevant for free‐ranging species in a field situation. Although it has to be acknowledged that our study animals do not represent a wild population and were not exposed to large thermal fluctuations for several years, our results nonetheless demonstrate the principle of our proposed model of thermal tolerance and changes in heat susceptibility.

**FIGURE 5:**
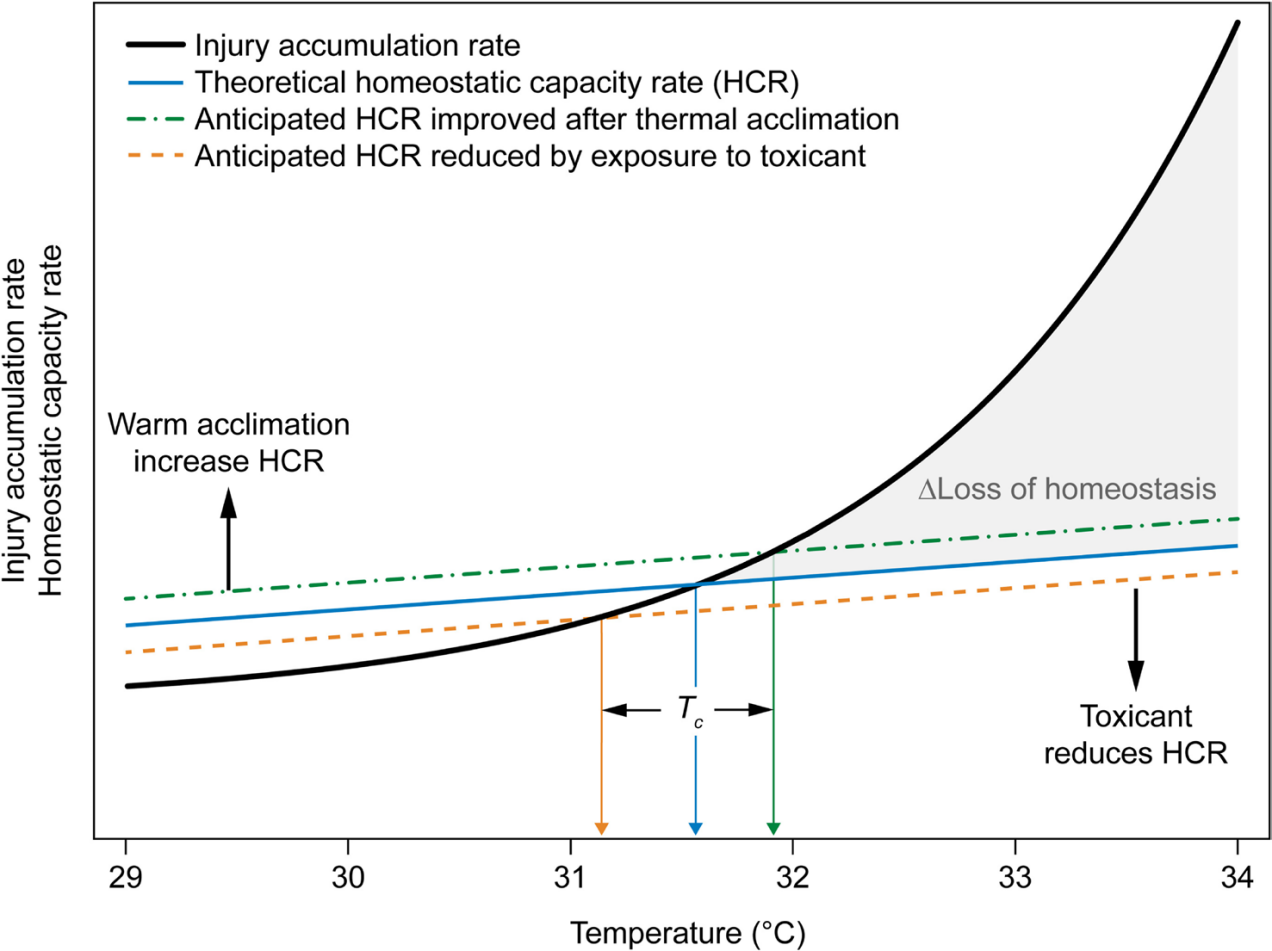
The thermal injury accumulation rate (solid black curve; the inverse of Lt_50_) is balanced by a capacity for cellular homeostatic maintenance (solid blue line). The homeostatic capacity rate (HCR) describes the ability of an organism to sustain and repair cellular damage by restoring and maintaining internal stability (homeostasis) in response to internal or external stressors. Ørsted et al. (2022) suggested a ~linear relationship of HCR with temperature. The black solid curve shows a theoretical relationship between rate of thermal injury accumulation and temperature. The blue solid line shows a theoretical homeostatic capacity rate under control conditions. When the temperature is below a critical temperature, *T*_c_ (indicated by the vertical blue arrow), the homeostatic capacity exceeds the injury accumulation rate, and the animal can maintain cellular homeostasis. At temperatures above *T*_c_, the rate of injury accumulation is higher than the homeostatic capacity rate, causing cellular damage to build up, followed by mortality (*ΔLoss of homeostasis*, the shaded area). In theory, exposure to a toxicant would decrease the homeostatic capacity rate since handling of the toxicant leaves less capacity to repair damage from thermal stress (shown as the orange dashed line). Similarly, acclimation to warmer conditions could improve the homeostatic capacity rate and with that the repair rate of thermal injury (shown as the dashed green line). Accordingly, *T*_c_ will be shifted to a higher temperature upon acclimation to warmer conditions (i.e., the animal becomes more tolerant of high temperatures), but to a lower temperature upon exposure to a toxicant (the animal becomes more susceptible to thermal stress). Lastly, the gain or loss of thermal tolerance will be much higher at relatively mild heat (i.e., slightly above *T*_c_) than at high thermal stress because the relative change in *Δloss of homeostasis* is considerably higher in the former case. The figure and its concept is adapted from Ørsted et al. (2022).

To explain this result, we have been inspired by a conceptual model of high‐temperature tolerance developed by Overgaard and co‐workers (Ørsted et al. 2022). These authors propose that the rate at which thermal injury accumulates in ectothermic organisms exposed to high temperature is a balance between the disruption of cellular homeostasis and the capacity to maintain cellular homeostasis (i.e., the ability to “repair” cellular injury) (Ørsted et al. 2022). A modification of this concept is outlined in Figure 5, which shows that the TDT we observed in our experiments reflects the net difference (ΔLoss of homeostasis) between the injury accumulation rate and the rate of homeostatic capacity. Recall that the injury accumulation rate is the inverse of Lt_50_and, hence, determining the exponential decay relationship between Lt_50_ and temperature shown in Figures 2 and 3. As an addition to this model, we here propose that the main effect of a toxicant administered to the test organism in sub‐lethal doses (as done in this study) will be manifested in a decreased cellular repair capacity (Figure 5). Contrary to this, acclimation to warmer conditions (in our study, acclimation to 24°C vs. 20°C) could increase heat tolerance by raising the homeostatic capacity rate without necessarily influencing the thermal injury accumulation rate (Figure 5). If this model holds true, we expect that the negative effects of toxicants and positive effects of warm acclimation are most conspicuous at temperatures slightly above *T*_c_ (here, at 32.5°C; Figure 4) and only small, if at all detectable, at very high temperatures (here, at 37°C; Figure 4). This is because the relative change in ΔLoss of homeostasis is considerably higher in the former case (Figure 5).

This expectation was met in the fluazinam treatments (acclimation at 20°C), showing that exposure to this toxicant caused a decrease in Lt_50‐32.5°C_ from 1100 to 800 min, whereas Lt_50‐37°C_ was unchanged. We observed the same tendency for copper‐exposed animals, but with a less clear effect. This suggests to us that the toxicants had overwhelmed the ability to repair thermal injury at moderately high temperatures, whereas injuries at high temperatures (37°C) accumulate so rapidly that repair mechanisms are relatively less influenced and become almost irrelevant.

We have not paired our experiment with molecular investigations to further explore the physiological mechanisms underlying ΔLoss of homeostasis and the origin of cellular injuries accumulating during heat exposure. From other studies, it has been suggested that heat injury develops through a combination of protein inactivation and denaturation, membrane dysfunction because of excessive fluidity of cellular membranes, and unbalanced temperature effects on interdependent metabolic reactions (Bowler 2018; Hochachka and Somero 2002; Neven 2000). Although our empirical data, in our view, substantiates the conceptual model shown in Figure 5, future research on the physiological mechanisms behind heat injury is warranted, especially in the case of multiple stressors as investigated here. To our knowledge, our TDT approach has not been applied in ecotoxicological studies before.

When we compare with previous TICS studies, we find that the present results are consistent with a previous study in which we saw that exposure to both copper in Hygum soil and fluazinam in LUFA soil caused higher mortality after long‐term exposure to temperatures close to *T*_c_ (Ge et al. 2024; Wehrli, Slotsbo, Fomsgaard, et al. 2024). Although springtails can efficiently regulate the internal concentration of copper (Hopkin 1989), it is likely that this ability comes with a cost because metal trafficking is energy‐demanding. Moreover, continuous exposure to elevated concentrations may likely induce oxidative stress, which can lead to the formation of excess free radicals and cellular injuries (Maria et al. 2014). Less is known about fluazinam, but the very high internal concentrations of hydroxyl‐fluazinam in exposed animals (Table 1) suggest that this toxicant would also influence the cellular homeostatic capacity. The increased conversion to the metabolite could lead to increased toxicity (Harwood et al. 2009). However, hydroxylfluazinam accumulating in soil as a result of microbial degradation may also have contributed to the high internal concentrations of this metabolite. In a previous study, we found a slightly increased concentration of hydroxylfluazinam in LUFA soil after 4 weeks (Wehrli, Slotsbo, Fomsgaard, et al. 2024), suggesting that the accumulation of the metabolite in *F. candida* is a result of both uptake from soil and detoxification processes.

Acclimation temperature had a significant and very clear effect on thermal tolerance. In a previous study using *F. candida* , we compared acclimation at a relatively low temperature (10°C) with acclimation at 20°C and found that both the slope of the TDT regression line and survival time at 32.5°C were much higher in the summer acclimated animals. In contrast, the upper critical temperature did not differ between the two acclimation groups (Wehrli, Slotsbo, Fomsgaard, et al. 2024). In the present study, we found that animals acclimated at 24°C performed better than animals acclimated at 20°C, even though 24°C is above the optimal temperature for body growth (Ge et al. 2023; Roeben et al. 2023; Wehrli, Slotsbo, Fomsgaard, et al. 2024). According to the model shown in Figure 5, the positive effect of acclimation at high temperatures on homeostatic capacity rate could cancel out the negative effect of a toxicant. Interestingly, we observed that TDT regression lines became similar when animals had been pre‐exposed to toxicants at 24°C, and the Lt_50‐32.5°C_ did no longer differ between exposure concentrations of fluazinam and copper, respectively (Figure 4). A similar pattern was observed for damselflies exposed to chlorpyrifos at stressful high temperatures. Chlorpyrifos reduced the heat tolerance of damselfly larvae; however, this was buffered by adaptation of damselflies to both increased mean temperature and increased daily fluctuations reaching extreme high temperature (Verheyen et al. 2019). Further, we did not find an elevated internal concentration of fluazinam, nor of hydroxyl fluazinam, at the higher acclimation temperature. Hence, these observations answered our second research question, suggesting that the positive effects of warm acclimation canceled out the negative effects of toxicants on thermal tolerance.

To our surprise, we saw that exposure to different soil types greatly impacted thermal tolerance. In our experiment, we started the pre‐exposure period with animals of the same size and age in all treatments, yet the animals clearly differed in their thermal tolerance. Unfortunately, we did not document the size or physiological condition of the animals at the end of the pre‐exposure period. The LUFA and the Hygum soils differed in several physico‐chemical properties, for example, in terms of pH, organic matter content, water retention, and so forth. Several studies have shown that soil type has an influence on the growth, reproduction, and development of *F. candida* (Martikainen 1996; van Gestel and Mol 2003). It is possible, or even likely, that the animals' physiology, development, and growth had adjusted differently to the two soil types during the pre‐conditioning period, evidently with consequences for their thermal tolerance. For now, we cannot identify any physiological explanation, but this observation calls for more research, which was beyond the scope of the present investigation.

Overall, our results suggest that thermal acclimation had a large influence on thermal tolerance in *F. candida* , followed by soil type. In comparison, the concentration of chemicals had a less significant influence on thermal tolerance than acclimation temperature and soil type, but more work is needed to explore this further. On the other hand, it is difficult to compare the chemical type since it was confounded by soil type.

In conclusion, the present study highlights that multiple stressor studies, including stressful high temperatures should include a full description of the TDT landscape in order to capture which combinations of stressors are critical and result in possible interactions predicted by the CITS or TICS framework. We also call for more research on the interaction between high extreme temperatures, acclimation effects, and the exposure to toxicants with molecular tools to better understand the homeostatic capacity rate (HCR) and generate more data on the cellular and physiological mechanisms underlying our findings.

## Author Contributions

**Micha Wehrli:** conceptualization, data curation, formal analysis, investigation, methodology, visualization, writing – original draft, writing – review and editing. **Jian Ge:** conceptualization, data curation, formal analysis, investigation, methodology, visualization, writing – original draft, writing – review and editing. **Stine Slotsbo:** conceptualization, data curation, formal analysis, supervision, writing – original draft, writing – review and editing. **Martin Holmstrup:** conceptualization, data curation, formal analysis, investigation, methodology, project administration, supervision, visualization, writing – original draft, writing – review and editing.

## Conflicts of Interest

The authors declare no conflicts of interest.

## Supporting information


Appendix S1.

## Data Availability

The data that support the findings of this study are openly available in Dryad at http://doi.org/10.5061/dryad.1rn8pk16m.
