# Supplementary material for: Pre‐Exposure to Chemicals Increases Springtail Vulnerability to High Temperatures
Source: Glob Chang Biol. 2025 Jul 23;31(7):e70374. doi: 10.1111/gcb.70374 (PMC12284914; doi:10.1111/gcb.70374)
Supplement: Supplementary file 1 — Appendix S1. [file GCB-31-e70374-s001.pdf]

Supplementary Information

Toxic heatwave: Chemical pre-exposure increases the heat failure rate of springtails

Micha Wehrli[a,b§], Jian Ge[a§], Stine Slotsbo[a] and Martin Holmstrup[a*]

[a] *Aarhus University, Department of Ecoscience, C.F. Møllers Allé 4, 8000 Aarhus C, Denmark*

[b] *Department of Environmental Chemistry, Swiss Federal Institute of Aquatic Science and Technology - Eawag, Überlandstrasse 133, CH-8600 Dübendorf, Switzerland*

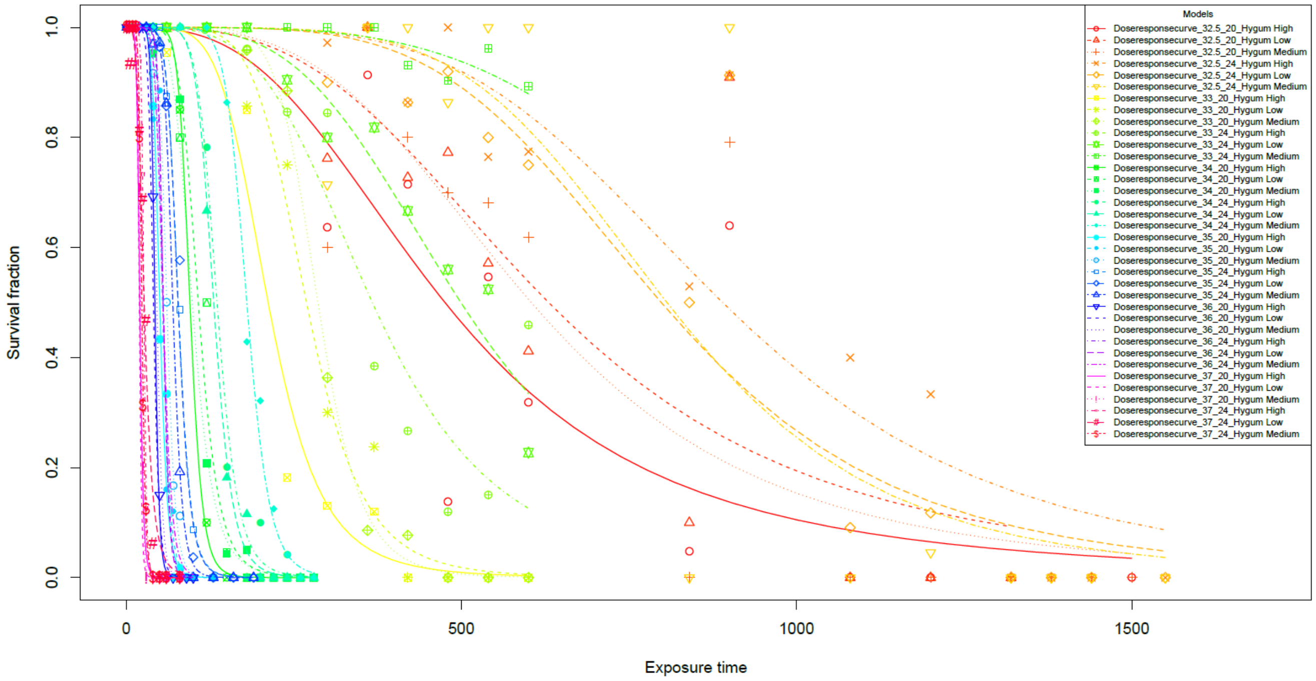

Figure S1A. Log-logistic fits of survival vs. exposure time of adult *Folsomia candida* pre-exposed in copper-polluted Hygum soil, and then exposed to a range of high temperatures.

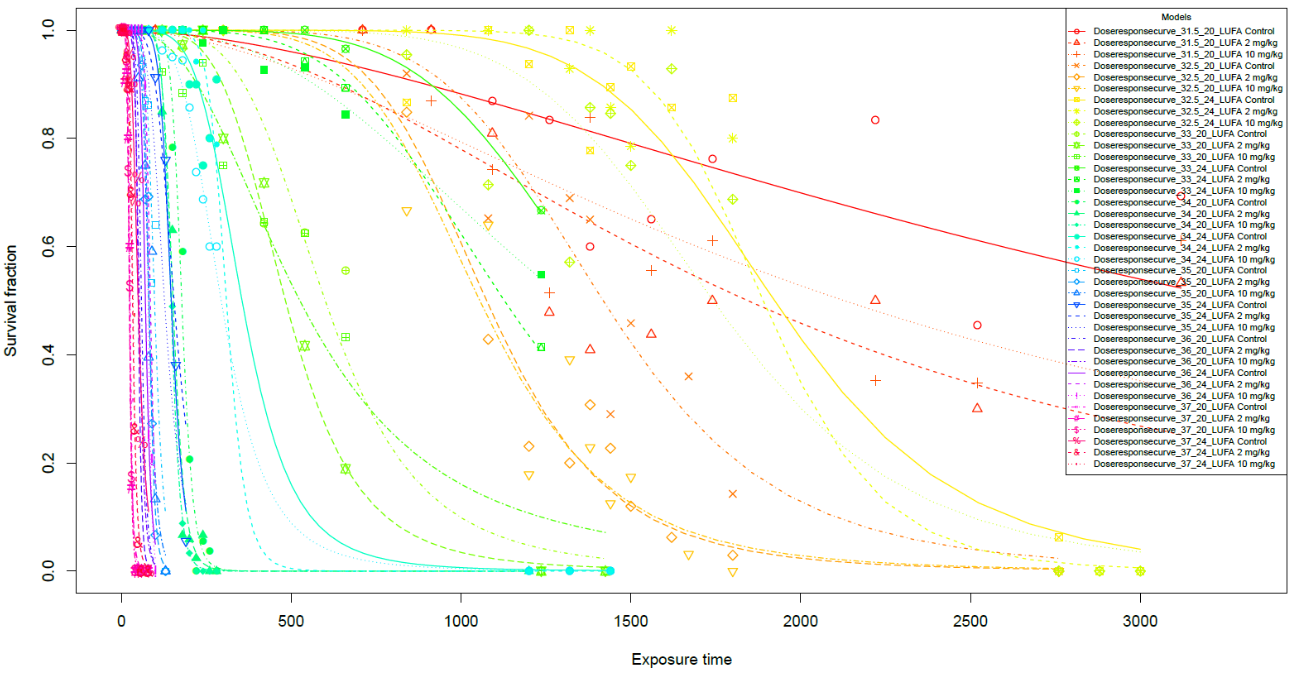

Figure S1B. Log-logistic fits of survival vs. exposure time of adult *Folsomia candida* pre-exposed in LUFA soil contaminated with fluazinam, and then exposed to a range of high temperatures.

Table S1A. Parameters of the bootstrap regression of Lt_{50} vs. temperature for *Folsomia candida* pre-exposed to copper-polluted Hygum soil.

Treatment	Log(Intercept)	Log(α)	LT50 at 32.5 °C (min)	LT50 at 37 °C (min)	Z	Q10
	20 °C population					
Control	12.76<br>(12.46, 13.01)	-0.31<br>(-0.32, -0.30)	427.98<br>(411.45, 447.02)	16.94<br>(16.31, 17.73)	3.21<br>(3.13, 3.30)	1306.99<br>(1065.94, 1549.02)
Medium	12.28<br>(12.03, 12.53)	-0.30<br>(-0.30, -0.29)	417.44<br>(403.38, 436.86)	19.23<br>(18.71, 20.04)	3.36<br>(3.28, 3.45)	937.23<br>(793.14, 1107.78)
High	11.56<br>(11.25, 11.87)	-0.28<br>(-0.29, -0.27)	328.16<br>(306.72, 347.15)	18.33<br>(17.91, 18.85)	3.59<br>(3.48, 3.71)	605.65<br>(497.93, 746.18)
	24 °C acclimation					
Control	13.17<br>(12.87, 13.46)	-0.32<br>(-0.33, -0.31)	619.73<br>(599.94, 642.64)	22.88<br>(21.64, 24.24)	3.13<br>(3.05, 3.22)	1555.99<br>(1276.08, 1901.85)
Medium	14.85<br>(14.00, 15.50)	-0.37<br>(-0.38, -0.34)	878.41<br>(714.24, 1034.47)	19.95<br>(18.99, 21.05)	2.73<br>(2.60, 2.91)	4603.75<br>(2695.88, 6952.92)
High	12.96<br>(12.76, 13.17)	-0.31<br>(-0.32, -0.31)	587.34<br>(563.65, 608.2)	22.94<br>(22.16, 23.80)	3.19<br>(3.13, 3.25)	1368.68<br>(1195.82, 1569.46)

Table S1B. Parameters of the bootstrap regression of Lt_{50} vs. temperature for *Folsomia candida* pre-exposed to LUFA soil contaminated with fluazinam.

Trt	Log(Intercept)	Log(α)	LT50 at 33 °C (min)	LT50 at 37 °C (min)	Z	Q10
20 °C population						
LUFA Control	15.41	-0.38	1088.82	20.88	2.63	6429.00
	(14.84, 16.04)	(-0.40, -0.36)	(976.28, 1186.36)	(19.69, 22.18)	(2.51, 2.74)	(4442.99, 9684.61)
LUFA 2 mg kg^{-1}	14.40	-0.35	771.44	19.66	2.82	3476.50
	(14.11, 14.67)	(-0.36, -0.35)	(735.35, 801.5)	(18.83, 20.47)	(2.76, 2.89)	(2895.43, 4165.27)
LUFA 10 mg kg^{-1}	14.59	-0.36	816.28	19.59	2.78	3931.82
	(14.26, 14.97)	(-0.37, -0.35)	(776.57, 857.86)	(18.7, 20.45)	(2.70, 2.86)	(3174.62, 5038.64)
24 °C acclimation						
Control	15.41	-0.37	1689.02	34.91	2.67	5586.04
	(15.06, 15.80)	(-0.39, -0.37)	(1550.44, 1815.12)	(33.35, 36.25)	(2.59, 2.74)	(4472.33, 7193.12)
LUFA 2 mg kg^{-1}	15.58	-0.38	1594.15	30.75	2.63	6423.48
	(15.28, 15.86)	(-0.39, -0.37)	(1531.53, 1703.25)	(30.05, 32.38)	(2.57, 2.69)	(5299.63, 7723.91)
LUFA 10 mg kg^{-1}	15.68	-0.38	1568.76	29.12	2.60	6938.36
	(15.22, 16.11)	(-0.40, -0.37)	(1391.57, 1702.2)	(28.1, 29.17)	(2.53, 2.69)	(5167.03, 9114.87)

[illegible]
